# Reviewed article: Chaves ACKA, Meucci RD. Monitoring influenza vaccination coverage among older adults: a rural cohort study, Rio Grande, 2017-2022. Epidemiol Serv Saude. 2026:35;e20250561.

**DOI:** 10.1590/S2237-96222026v35e20250561.b

**Published:** 2026-03-23

**Authors:** João Paulo Cola

**Affiliations:** 1 Prefeitura Municipal de São Mateus, São Mateus , ES, Brazil

## Peer review B

## Questionnaire

Does the manuscript contain new and significant information to justify publication? Yes

Does the Abstract (Summary) clearly and accurately describe the content of the article? No

Is the problem significant and concisely stated? Yes

Are the methods described comprehensively? Yes

Are the interpretations and conclusions justified by the results? Yes

Is adequate reference made to other work in the field? Yes

## Manuscript Structure

Length of article is: Adequate

Number of tables is: Adequate

Number of figures is: Adequate

Please state any conflict(s) of interest that you have in relation to the review of this paper. None

Interest: Excellent

Quality: Good

Originality: Excellent

Overall: Good

Recommendation: Major Revision

## Comments

O manuscrito em avaliação apresenta um estudo de coorte com o objetivo de estimar a cobertura vacinal contra a influenza e identificar fatores associados à trajetória de vacinação entre pessoas idosas participantes da coorte EpiRural, no município de Rio Grande (RS). Os autores relatam um aumento progressivo da cobertura vacinal ao longo do seguimento, alcançando 85,7% ao final do período. A vacinação completa mostrou associação com características como não exercer atividade laboral, histórico de tabagismo (ex-fumantes ou nunca fumantes), presença de diagnóstico autorreferido de enfisema pulmonar, utilização de serviços de saúde no último ano e uso contínuo de medicamentos.

O estudo aborda uma temática relevante e pouco explorada nas análises nacionais de cobertura vacinal. Os resultados apresentados podem contribuir para o desenvolvimento de estratégias de busca ativa, ações de comunicação em saúde e fortalecimento da atenção primária, com foco na promoção da equidade no acesso à imunização entre pessoas idosas residentes em áreas rurais. No entanto, observo que o manuscrito requer ajustes substanciais em diferentes dimensões, conforme descrito a seguir:

Comentários críticos e importantes aos autores - correções maiores:

1. Revisar a descrição da análise estatística. O modo como a análise múltipla foi conduzida e como se deu o ajuste do modelo apresenta-se confuso e pouco esclarecido no manuscrito. Não está claro se os autores utilizaram um modelo tradicional de ajuste ou uma abordagem hierarquizada. Caso tenham adotado um modelo hierárquico, é imprescindível descreverem de forma clara os critérios para definição dos níveis, bem como o processo de ajuste entre eles, indicando quais variáveis foram incluídas em cada etapa.

2. Observa-se uma possível colinearidade entre as variáveis incluídas no modelo que se mostraram estatisticamente significativas em relação à variável dependente. No entanto, não está claro se essa colinearidade foi devidamente avaliada e controlada.

3. Considerando que os autores utilizaram a regressão logística ordinal para estimar as razões de chances, recomendo que incluam uma descrição sucinta sobre a interpretação da medida de associação obtida nesse modelo. Por tratar-se de uma variável ordinal e não binária, é importante explicitar como se dá a comparação entre as categorias da variável dependente e qual o significado das razões de chances estimadas.

4. Aprofundar a discussão sobre o viés de aferição relacionado à variável de interesse, uma vez que a informação sobre a vacinação contra influenza foi obtida por meio de autorrelato. Embora os autores reconheçam essa limitação, incluir uma análise crítica do impacto desse viés contribuirá para maior clareza na interpretação das estimativas de cobertura vacinal.

5. Descrever de forma mais detalhada a amostragem da coorte. O manuscrito não traz informações básicas de como o cálculo foi realizado o para a coorte original. Se há um estudo já publicado que apresente a amostragem da coorte, é recomendável citar. Além disso, considerando as perdas significativas ao longo do seguimento, seria essencial que os autores apresentassem uma estimativa do poder estatístico ou um cálculo amostral específico para o objetivo atual.

6. Ampliar a discussão sobre os cenários contrastantes de alcance e não alcance da cobertura vacinal identificados no estudo. Os dados sugerem realidades heterogêneas entre os contextos analisados, mas essa heterogeneidade não é suficientemente debatida, bem como a cobertura vacinal encontrada é abaixo da meta estabelecida pelo Ministério da Saúde.

7. Aprofundar a discussão dos fatores associados à cobertura vacinal observados no estudo, principalmente os fatores relacionados a questões respiratórias (tabagismos e enfisema pulmonar), relacionados ao cuidado longitudinal da atenção primária a saúde (uso de medicação continua e assistência a saúde no último ano).

8. Revisar o resumo do manuscrito para assegurar que as conclusões apresentadas não extrapolem os achados do estudo e que estejam alinhadas com o objetivo proposto. Além disso, sugere-se uma reavaliação da forma como os resultados são descritos, uma vez que algumas sentenças interpretam os dados, atribuindo significados que não estão plenamente sustentados pelas análises realizadas.

Comentários desejáveis aos autores - correções menores:

1. Rever a utilização do termo prevalência. Observa-se que os autores utilizam o termo “prevalência” para se referir as estimativas de cobertura vacinal em um estudo de coorte. Conceitualmente, prevalência é um termo mais associado a delineamentos transversais e não é convencional utilizá-lo para descrever estimativas em estudos de coorte. Recomendo substituir expressões como “prevalência da cobertura vacinal” por simplesmente “cobertura vacinal”, para maior precisão e clareza metodológica.

2. Revisar o objetivo do estudo apresentado no manuscrito. Observa-se que o objetivo descrito no corpo do texto não contempla explicitamente a análise dos fatores associados à trajetória de vacinação, diferentemente do que consta no resumo.

3. No Gráfico 1, os autores poderiam apresentar de forma mais detalhada a distribuição da cobertura vacinal por dose em cada ponto de seguimento da coorte. Sugere-se que informem, por exemplo, no primeiro acompanhamento, o número de idosos sem nenhuma dose, com uma dose e com duas doses; e, no segundo acompanhamento, a quantidade de indivíduos com nenhuma, uma, duas ou três doses. Essa apresentação tornaria mais clara e representativa a evolução da cobertura vacinal ao longo do tempo.

4. Avaliar a inclusão das frequências absolutas na Tabela 2, juntamente com as frequências relativas já apresentadas. A apresentação dos valores absolutos confere maior transparência aos dados.

5. Reorganizar a tabela 3. Não fica claro na tabela a distribuição das variáveis conforme os níveis hierárquicos mencionados na metodologia. Recomenda-se que os autores reorganizem a tabela de modo a refletir explicitamente a estrutura hierarquizada adotada na análise, indicando os blocos ou níveis em que cada variável se insere.

6. Rever a terminologia das variáveis “trabalha, álcool, hipertensão, diabetes, Aten. no posto de Saúde no últ. ano”, principalmente nas tabelas. Se faz necessário padronizar a terminologia em todo o manuscrito.

7. Evitar termos estigmatizantes conforme boas práticas editoriais internacionais. Sugiro utilizar o termo pessoa idosa em todo manuscrito.

8. Avaliar a pertinência de incluir o descritor “Cobertura Vacinal”, já existente no DeCS, entre as palavras-chave do manuscrito.

